# Construction of a novel step-scheme CdS/Pt/Bi_2_MoO_6_ photocatalyst for efficient photocatalytic fuel denitrification†

**DOI:** 10.1039/d1ra04417f

**Published:** 2021-07-01

**Authors:** Weineng Hu, Guiyang Yan, Ruowen Liang, Mengmeng Jiang, Renkun Huang, Yuzhou Xia, Lu Chen, Yi Lu

**Affiliations:** Province University Key Laboratory of Green Energy and Environment Catalysis, Ningde Normal University Ningde 352100 Fujian China; Fujian Provincial Key Laboratory of Featured Materials in Biochemical Industry, Ningde Normal University Ningde 352100 Fujian China ygyfjnu@163.com; State Key Laboratory of Photocatalysis on Energy and Environment, Fuzhou University Fuzhou 350002 P. R. China

## Abstract

Construction of step-scheme (S-scheme) heterojunction (HJ) structures is an excellent strategy to achieve efficient photogenerated carrier separation and retain strong redox ability. Recently, the development of efficient S-scheme HJ photocatalysts for the degradation of environmental organic pollutants has attracted considerable attention. In this work, a novel S-scheme CdS/Pt/Bi_2_MoO_6_ (CPB) photocatalyst was prepared for the first time by sonochemical and solvothermal methods. By anchoring Pt nanoparticles (NPs) at the interface between CdS nanorods (NRs) and Bi_2_MoO_6_ nanosheets (NSs), the migration of photogenerated electron–hole pairs along the stepped path was achieved. The ternary CPB samples were characterized by various analytical techniques, and their photocatalytic performance was investigated by conducting simulated fuel denitrification under visible-light irradiation. It was found that the CPB-4 composites exhibited the highest pyridine degradation activity, which reached 94% after 4 h of visible-light irradiation. The superior photocatalytic performance of the CPB-4 composite could be attributed to the synergistic effect of the Pt NPs and Bi_2_MoO_6_ NRs on the photocatalytic degradation as well as to the introduction of Pt and Bi_2_MoO_6_, which led to an excellent response and large specific surface area of the CPB-4 composite. Lastly, the bridging role of the Pt NPs introduced into the S-scheme system was also notable, as it effectively improved the separation and transfer of the CdS/Bi_2_MoO_6_ interfaces for the photogenerated electron–hole pairs while retaining strong redox ability.

## Abstract 2

The S-scheme heterojunction in which Pt nanoparticles were anchored between CdS and Bi_2_MoO_6_ as ‘bridges’ enhanced the utilization of visible light and efficient separation of photogenerated electron–hole pairs.

## Graphical abstract

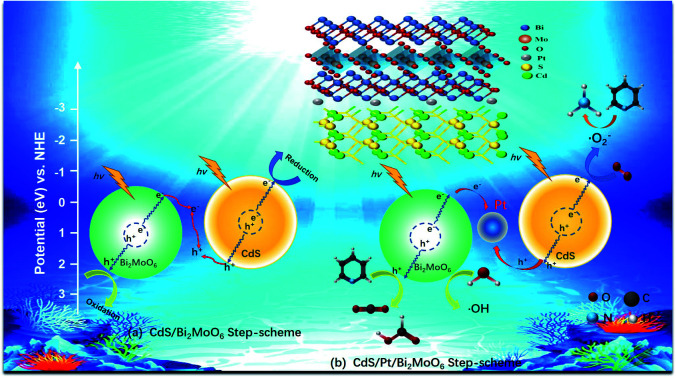

## Introduction

It is widely known that petroleum is the most utilized energy source in the world. Nevertheless, its use is associated with serious environmental problems.^1,2^ Numerous nitrogen-containing compounds (NCCs) and sulfur-containing compounds are produced during processes employing petroleum.^3^ The negative effects of NCCs include the formation of photochemical smog and acid rain, destruction of the ozone layer, and significant damage to human and animal health as well as the ecosystem. The removal of NCCs from gasoline is typically conducted by catalytic hydrogenation.^4^ However, this method requires high operating temperatures and pressures. Hence, the development of economical and environmentally friendly approaches for the removal of NCCs from petroleum is highly desired.

Previous studies confirmed that the photocatalytic technology could be used to effectively solve the issues associated with NCCs.^5–8^ For example, Liang *et al.*^9^ prepared sodium dodecyl sulfate (SDS)-modified metal–organic framework (MOF)-derived porous Fe_2_O_3_ nanoparticles (NPs). The generated NPs exhibited high catalytic activity and good recyclability in fuel denitrification. After 240 min of visible-light irradiation (*λ* ≥ 420 nm), the prepared photocatalyst showed nearly 100% fuel denitration rate. Moreover, Meng *et al.*^10^ reported electrospun nanofibers of Pd-doped α-Bi_2_O_3_, which degraded up to 84.9% of pyridine after 60 min of visible-light radiation. Among the traditional photocatalysts considered in the current research, CdS is a typical reduced photocatalyst, which is widely employed in H_2_ production^11^ and organic pollutant degradation.^12^ Regrettably, there are several drawbacks of using CdS in photocatalytic processes. First, CdS has a narrow band gap (2.4 eV), enabling a wide visible-light response;^13^ However, the rapid recombination rate of photo-induced electron–hole pairs in the CdS bulk phase severely reduces its photocatalytic activity.^14^ Second, CdS is usually composed of large particles, which leads to the formation of limited active sites and low photocatalytic efficiency.^15^ Hence, the development of an effective strategy to improve the efficiency of the photogenerated electron–hole pair separation in CdS is essential. Several approaches to overcome the above challenges have been reported. These include metal deposition,^16,17^ heterojunction (HJ) design,^18^ and semiconductor coupling.^19^ For instance, Hu *et al.*^20^ designed Bi_2_MoO_6_/CdS step-scheme (S-scheme) HJ composites, which displayed excellent photocatalytic activity under visible light. Fang *et al.*^21,22^ fabricated hierarchical CuO–TiO_2_ hollow microsphere composites for highly efficient photodrive CO_2_ reduction. Furthermore, Yu *et al.*^23^ developed a novel B-doped BiOCl PC exposed to the (001) plane, which showed remarkable performance in the degradation of organic pollutants. The assembly and construction of these three-dimensional materials greatly promoted the exposure of reverse active sites and improved the photocatalytic or photoelectrocatalytic activity.^24–26^

Nevertheless, the improvement of the separation efficiency of the photogenerated electron–hole pairs by conventional HJ schemes typically comes at the cost of reducing the redox capabilities of composite semiconductors.^27,28^ Photogenerated electron–hole pairs must transfer from high to low potentials to achieve separation. However, pollutant degradation reactions or organic synthesis processes usually require a certain redox potential, which is unfavorable for photocatalytic reactions. Yu *et al.*^29^ evaluated S-scheme HJ photocatalysts to improve their redox ability. Additionally, the mechanism of the photogenerated charge transfer was elucidated. Moreover, Zhong *et al.*^30^ developed BiVO_4_@MoS_2_ core–shell S-scheme HJ by solvothermal technique, which completely degrades RhB solution within 20 minutes. Dai *et al.*^31^ reported a novel S-scheme BiVO_4_/Ag_3_VO_4_ photocatalyst that exhibited excellent photocatalytic activity for the degradation of methyl blue (MB) under visible-light irradiation.

Bismuth molybdate (Bi_2_MoO_6_), a member of the aurivillius family of layered perovskites, is composed of alternating stacks of [Bi_2_O_2_]^2+^ and [MoO_4_]^2−^ layers. It has recently attracted significant attention due to its narrow band gap (2.5–2.7 eV) in response to visible light.^32,33^ Hence, numerous studies involved coupling of Bi_2_MoO_6_ with other semiconductor materials to achieve efficient photogenerated carrier transfer and separation. The previously reported materials include TiO_2_/Bi_2_MoO_6_,^34^ MoS_2_/Bi_2_MoO_6_,^35^ Bi_2_S_3_/Bi_2_MoO_6_,^36^ AgI/Bi_2_MoO_6_,^37^ and C_3_N_4_/Bi_2_MoO_6_.^38^ Xiu *et al.*^39^ loaded silver phosphate nanocrystals on a spherical Bi_2_MoO_6_ surface by a deposition–precipitation technique to achieve good photocatalytic performance for the degradation of RhB and MB. In addition, Zhang *et al.*^40^ prepared a flower-like Ag_2_O/Bi_2_MoO_6_ p–n HJ by a lysosomal thermal deposition–precipitation method. The developed photocatalyst retained strong redox ability. Xiao *et al.*^41^ constructed a Z-scheme system utilizing the synergistic effects of Ag, g-C_3_N_4_, and Bi_2_WO_6_ nanostructures to achieve rapid electron–hole separation, which resulted in outstanding photocatalytic activity. Meanwhile, Bi_2_MoO_6_ is an oxidized semiconductor photocatalyst with a large work function, while CdS is a reduced semiconductor photocatalyst with a small work function. It has been shown that Bi_2_MoO_6_ and CdS exhibit excellent band structures, which enable the construction of S-scheme HJs.

Based on the above studies, we speculated that S-scheme HJ photocatalysts composed of Bi_2_MoO_6_, CdS, and Pt would enable efficient separation of photogenerated electron–hole pairs while retaining a strong redox ability and the three species combined greatly increased the specific surface area and reaction sites of the PC.^42,43^ Pt NPs could act as electron “bridges” to induce the transfer of electron–hole pairs along the S-scheme migration path as well as to improve the photocatalytic activity. In this study, we developed a novel S-scheme HJ ternary CdS/Pt/Bi_2_MoO_6_ (CPB) composite, which was utilized as a photocatalyst in the denitrification of fuel under visible-light irradiation. By controlling the mass ratio of Pt and CdS, the loading of Pt on the surface of CdS nanorods (NRs) was effectively adjusted. It was found that 0.5 wt% Pt/CdS NRs showed the highest light absorption capacity and photocatalytic fuel denitrification activity. We also modified the loading of Bi_2_MoO_6_ on the surface of Pt/CdS, which significantly affected the photogenerated electron–hole recombination efficiency as well as the photocatalytic activity of the prepared CPB photocatalyst. The experimental results revealed that ternary CPB-4 composites exhibited the strongest photocatalytic performance and favorable cycling stability under visible-light irradiation. Additionally, we elucidated the mechanism of the prepared S-scheme HJ by Kelvin probe force microscopy and electron spin resonance (ESR) analyses. Lastly, the reasons for the enhanced photocatalytic fuel denitrification activity were investigated.

## Experimental

### Materials and reagents

Bismuth nitrate (Bi(NO_3_)_3_·5H_2_O), sodium molybdat (Na_2_MoO_4_·2H_2_O), cadmium acetate (Cd(CH_3_CO_2_)_2_·2H_2_O), thiourea (CH_4_N_2_S), ethylenediamine (EDA), ethylene glycol (EG), chloroplatinic acid hexahydrate (H_2_PtCl_6_·6H_2_O, AR) were purchased from Sinopharm Chemical Reagent Co. Ltd. All chemicals and reagents were used as received from commercial suppliers without further purification, deionized water was used in all experiments.

### Synthesis of 1D CdS NRs

Pure 1D CdS NRs were synthesized by the one-step solvothermal method.^44^ 0.853 g of Cd(CH_3_CO_2_)_2_·2H_2_O was dissolved in 60 mL of EDA with vigorous magnetic stirring for 10 min. Then, 1.216 g CH_4_N_2_S was added to the mixed solution and stirred vigorously for 120 min at room temperature, and the mixed solution was kept in a relatively sealed environment. The solution was transferred to a polytetrafluoroethylene-lined stainless steel autoclave, heated to 160 °C and kept for 48 h. After cooling to room temperature, the yellow precipitate was separated by centrifugation, washed with deionized water and ethanol alternately several times, and finally dried in a vacuum oven at 60 °C overnight.

### Synthesis of Pt/CdS

The Pt/CdS was prepared by ultrasonic chemical deposition.^45^ In general, 0.200 g of 1D CdS NRs was dispersed into a self-made quartz glass tube containing 25 mL of the aqueous solution, and then 10 mL of methanol was added for vigorous stirring. The prepared H_2_PtCl_6_·6H_2_O (*x* wt% Pt *vs.* CdS) was added into the mixture according to the required amount, and then nitrogen was introduced into the dispersion for continuous bubbling for 1 h until dissolved oxygen was discharged. The quartz glass tube was completely immersed in the ultrasonic cleaning tank (40 kHz, 150 W) for ultrasonic chemical deposition of Pt on 1D CdS NRs surface. After 30 min of ultrasonic irradiation, the product was collected by suction filtration and cleaned alternately with deionized water and ethanol. Finally, the obtained product was dried overnight in a vacuum oven at 60 °C. 0.5, 1.0, and 1.5 wt% Pt/CdS NRs were obtained according to the amount of H_2_PtCl_6_·6H_2_O.

### Synthesis of CdS/Pt/Bi_2_MoO_6_ photocatalysts

**Scheme 1:**
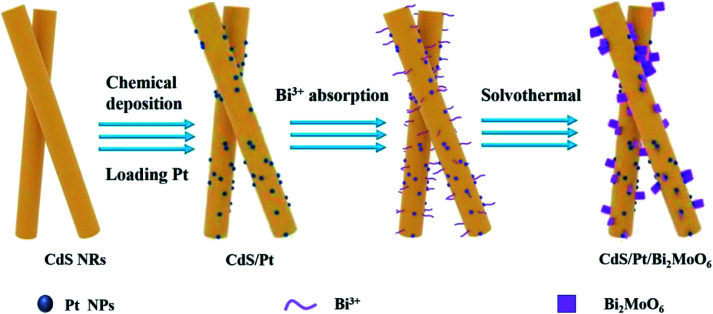
Schematic of CdS/Pt/Bi_2_MoO_6_ composites synthesis.

All the CdS/Pt/Bi_2_MoO_6_ PCs were synthesized by solvothermal reactions, as shown in Scheme 1. First, a certain mass of Bi(NO_3_)_3_·5H_2_O was dissolved into 6 mL EG to form a clear solution by ultrasound. 0.145 g 0.5 wt% Pt/CdS NRs catalyst was dispersed in the mixture, 30 mL ethanol was added and stirred in the dark for 2 h to achieve the preferential adsorption of Bi^3+^ on Pt/CdS NRs. Subsequently, the corresponding amount of Na_2_MoO_4_·2H_2_O was added as the precursor of molybdenum source and continued stirring for 0.5 h. Finally, the above-mixed solution was transferred into a 50 mL polytetrafluoroethylene-lined stainless steel autoclave and heated at 160 °C for 24 h. After cooling to room temperature, the yellow-green precipitate was separated by centrifugation, washed with deionized water and ethanol alternately several times, and finally dried in a vacuum oven at 60 °C for one night to obtain CdS/Pt/Bi_2_MoO_6_ (CPB). The CPB composites with a molar ratio of Bi_2_MoO_6_ to Pt/CdS NRs of 10, 20, 30, 40, 65, and 100% were marked as CPB-1, CPB-2, CPB-3, CPB-4, CPB-5, CPB-6, respectively. As a comparison, CdS/Bi_2_MoO_6_ composites without Pt loading was prepared by replacing CdS/Pt with pure CdS under the same conditions.

### Characterization

The crystal structures of all samples were characterized by X-ray powder diffraction (XRD). The XRD patterns were obtained on Bruker D8 advance X-ray diffractometer irradiated with Cu Kα (*λ* = 0.15406 nm) at 40 kV and 40 mA. The scanning angle is 10–80° in the range of 2*θ*. The morphology and microstructure of all samples were characterized by scanning electron microscopy (SEM, Hitachi S-4700) and transmission electron microscopy (TEM, JEOL JEM 2100F). Ultraviolet-visible diffuse reflectance spectra (UV-Vis DRS) were obtained using a Shimadzu UV-2700 UV-Vis spectrophotometer. White solid barium sulfate powder was used as the standard reference, and the scanning range was 800–250 nm. The Brunauer–Emmett–Teller (BET) surface area of all samples was measured by ASAP 2460 apparatus (Micromeritics Instrument Corp., USA). X-ray photoelectron spectroscopy (XPS) measurements were carried out using a Thermo Scientific K-Alpha^+^ spectrometer with Al-Kα radiation to obtain the surface elemental composition of all samples. The binding energies of all samples were corrected by C 1s binding energy of 284.8 eV. The electron spin response (ESR) experiment was carried out with a Bruker A300 spectrometer. Spin trapping ESR signals were recorded with 5,5-dimethyl-1-pyrroline-*n*-oxide (DMPO) and 2,2,6,6-tetramethyl-1-piperidinyloxy (TEMPO) at visible wavelengths above 420 nm. All photocatalytic degradation products were analyzed by high-performance liquid chromatography-mass spectrometry (HPLC-MS). Agilent 1200 system was used with Agilent Zorbax eclipse xdb-c18 column (2.1 mm × 100 mm × 3.5 mm). The photoluminescence spectra (PL) of all samples were tested by fluorescence spectrometer (FLS 980). The powder sample is placed in the corresponding quartz device to test with solid test accessories. The test condition is room temperature, the excitation light source is xenon lamp, the excitation wavelength is 340 nm, and the detector is a photomultiplier tube. The electrochemical measurements were performed by an electrochemical workstation CHI-660D in a three-electrode cell, Ag/AgCl electrode was used as the reference electrode and a Pt plate was used as the counter electrode. Mott–Schottky measurements were performed using a Zahner Zennium electrochemical workstation with the potential ranged from −1.5 to 1 V in which calomel electrodes are used as reference electro. The work functions of PCs were characterized by Kelvin probe. The Au probe was calibrated by the Au block, followed by the work function of the catalyst in the dark, in which 800 points were collected.

### Photocatalytic activity testing

The photocatalytic activity of CPB composites was studied by photocatalytic denitrification of pyridine under visible light (*λ* ≥ 420 nm). The 150 μg g^−1^ simulated NCCs-containing gasoline fuel was prepared by dissolving pyridine in 1.0 L *n*-octane. Then, 50 mg catalyst and 50 mL pyridine/octane solution were added into a specially made quartz glass reactor with magnetic stirring. The dispersion was stirred in the dark for 2 h to establish the adsorption–desorption equilibrium. Subsequently, the dispersion was irradiated with a 300W xenon lamp (PLS-SXE 300, Beijing Perfectlight Co. Ltd), which was equipped with an ultraviolet cutting filter to cut off light with a wavelength less than 420 nm. Then an aliquot of the solution sample (2 mL) was taken every half hour, and the suspension was centrifuged. A Varian Cary50 spectrometer was used to detect the residual concentration of pyridine in the supernatant at the peak position of 251 nm.

## Results and discussion

**Fig. 1:**
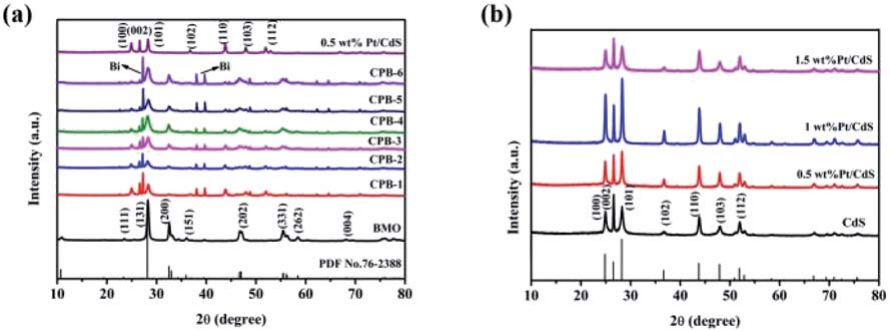
XRD patterns of (a) CdS NRs, Bi_2_MoO_6_ NSs, 0.5 wt% Pt/CdS, and a series of ternary CPB composites (b) a series of Pt/CdS composites.

The crystal structures and purity of the prepared samples were assessed by XRD. Fig. 1 shows that the characteristic peaks of 1D CdS NRs and binary Pt/CdS were consistent with a hexagonal system of CdS.^45^ However, no obvious diffraction peaks corresponding to metallic Pt NPs were observed in the XRD spectrum of binary Pt/CdS, which could be attributed to the uniform distribution of a small amount of Pt. Meanwhile, as shown in Fig. 1a, the XRD pattern of pure Bi_2_MoO_6_ exhibited distinct diffraction peaks at 2*θ* of 28.3°, 32.53°, 46.74°, 55.44°, and 58.48°, which were ascribed to the (131), (002), (202), (331), and (262) crystal planes of orthorhombic Bi_2_MoO_6_ (JCPDS 76-2388),^46^ respectively. The peaks corresponding to hexagonal phase CdS and orthorhombic Bi_2_MoO_6_ were detected in the diffractograms of all ternary CPB photocatalysts with good crystallinity. Nonetheless, in the presence of both CdS NRs and Pt NPs, a small amount of the Bi monomer was reduced. This phenomenon was not observed in the presence of just CdS NRs (Fig. S1†). Overall, Bi_2_MoO_6_ nanostructures were successfully synthesized on Pt/CdS by a solvothermal method.

**Fig. 2:**
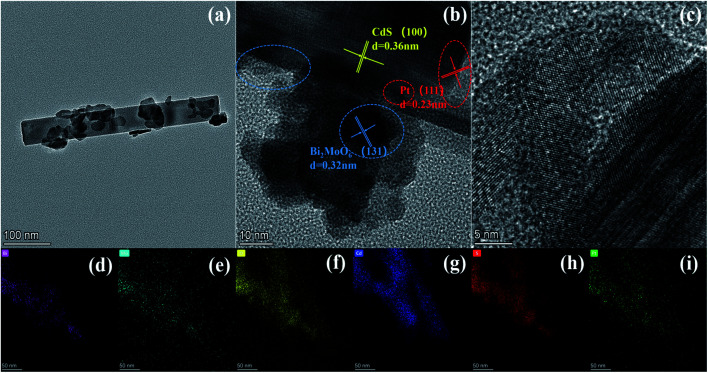
Typical TEM images of (a) CPB-4, (b and c) HRTEM images of CPB-4 and (d–i) element mapping images of CPB-4.

We also analyzed the morphology and microstructure of 0.5 wt% Pt/CdS and ternary CPB-4 composites by TEM and HRTEM. As shown in Fig. S2a,† in 0.5 wt% Pt/CdS, CdS exhibited a rod-like structure after ultrasonic irradiation. Moreover, small, dark gray Pt NPs were highly deposited on the surface of the CdS NR substrate. Fig. S2b and c† shows the HRTEM images of CdS NRs and Pt NPs, respectively. HTEM analysis revealed the incorporation of metallic Pt NPs on the well-crystallized CdS surface. The measured lattice fringes of 0.360 nm corresponded to the hexagonal CdS (100) crystal plane, while those of 0.230 nm were ascribed to the crystal plane of cubic phase Pt (111).^47^ The HAADF-STEM mapping analysis (Fig. S2c–g†) of the selected 0.5 wt% Pt/CdS NRs showed the uniform distribution of S, Cd, and Pt. In addition, as demonstrated in Fig. 2a, the ternary CPB-4 sample was a HJ structure, in which the Pt/CdS NRs were covered with Bi_2_MoO_6_ NSs. The results of this analysis revealed the formation of a ternary CPB HJ. The lattice fringe spacing was established at 0.315 and 0.360 nm, which corresponded to the (131) crystal plane of Bi_2_MoO_6_ (ref. 46) and the (100) crystal plane of CdS, respectively. Furthermore, the (111) plane of Pt with a lattice fringe of 0.230 nm was clearly observed at the interface of the Bi_2_MoO_6_ NSs with the CdS NRs, indicating the successful construction of CdS/Pt/Bi_2_MoO_6_ HJ composites. Fig. 2d–i shows the presence of Bi, Mo, O, Cd, S, and Pt in the ternary CPB-4 sample, which was consistent with the EDS analysis (Fig. S3†). These results further demonstrated the effective loading of Bi_2_MoO_6_ onto the Pt/CdS NRs.

**Fig. 3:**
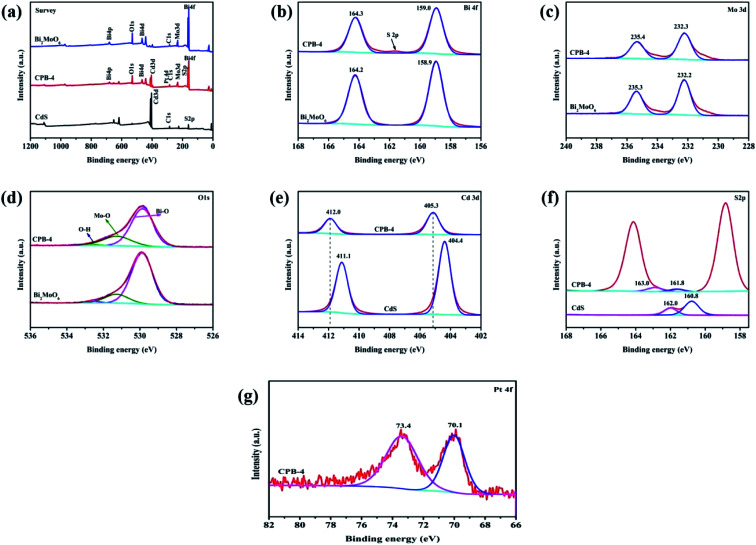
XPS analysis of CPB-4 and Bi_2_MoO_6_ showing (a) survey, (b) Bi 4f, (c) Mo 3d, (d) O 1s, (e) Cd 3d, (f) S 2p, and (g) Pt 4f spectra.

**Fig. 4:**
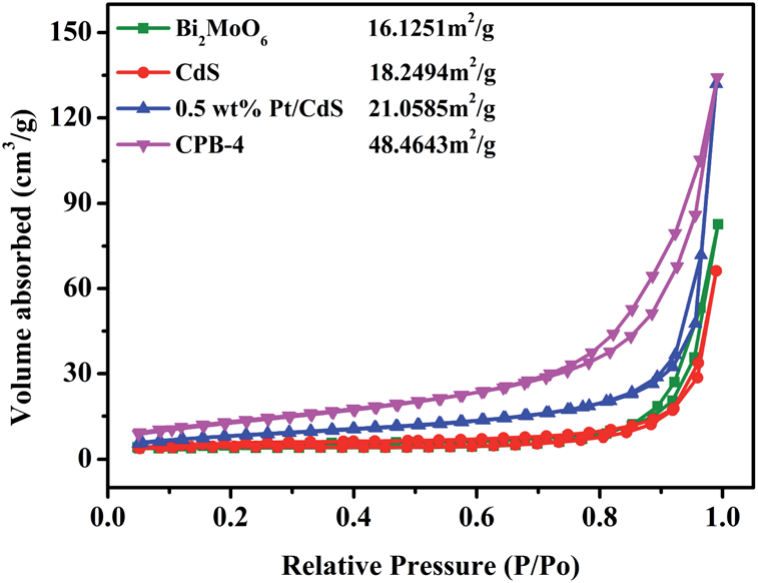
N_2_ adsorption–desorption isotherms of CdS, Bi_2_MoO_6_, Pt/CdS, and CPB-4 composites.

We subsequently acquired XPS spectra of CdS, Bi_2_MoO_6_, and ternary CPB-4 to investigate the surface valence states and chemical compositions of the samples. As shown in Fig. 3a, CPB-4 contained Bi, Mo, O, Cd, S, and Pt, which was in agreement with the elemental analysis. Additionally, the presence of C was ascribed to the contamination of the instrument. Fig. 3b–g illustrates the high-resolution XPS spectra of Bi 4f, Mo 3d, O 1s, Cd 3d, S 2p, and Pt 4f, respectively. As shown in Fig. 3b, the spectrum of Bi_2_MoO_6_ exhibited two peaks at 158.9 and 164.2 eV, which corresponded to the Bi 4f_7/2_ and Bi 4f_5/2_ orbitals, respectively. Moreover, as demonstrated in Fig. 4c, the two intense peaks of Bi_2_MoO_6_ at 232.2 and 235.3 eV were ascribed to Mo 3d_5/2_ and Mo 3d_3/2_ of Mo^6+^,^48^ respectively. Compared with Bi_2_MoO_6_, the Bi 4f and Mo 3d peaks of CPB-4 exhibited almost no shift. As can be seen in Fig. 3d, the spectra of Bi_2_MoO_6_ and CPB-4 displayed three asymmetric O 1s peaks at 529.9, 531.3, and 532.6 eV, which were assigned to Bi–O, Mo–O, and H–O,^46^ correspondingly. The spectrum of pure CdS (Fig. 3e) showed two peaks at 404.4 and 411.1 eV, which were attributed to Cd 3d_5/2_ and Cd 3d_3/2_ tracks,^49^ respectively. Compared with CdS, the Cd 3d peaks of CPB-4 shifted to 405.3 and 412.0 eV, respectively, while the peak spacing was identical to that of Cd 3d peaks in CdS (0.9 eV). The detected shift was attributed to the effect of the Fermi level equilibrium. In the ternary CPB-4 composite photocatalyst, the electrons migrated from CdS to the Pt NPs to reach the Fermi level equilibrium, which resulted in a 0.9 eV shift in the binding energy of Cd 3d. The binding energy shift in CPB-4 also reflected the presence of a well-controlled interface between the CdS NRs and Pt NPs. The high-resolution spectrum of S 2p of CdS (Fig. 3f) revealed two peaks at 160.8 and 162.0 eV, which corresponded to S 2p_3/2_ and S 2p_1/2_ in the form of S^2−^, respectively. The S 2p peaks of CPB-4 shifted to 161.8 and 163.0 eV due to the combination of ternary Bi_2_MoO_6_ and Pt/CdS. As illustrated in Fig. 4g, in the XPS spectrum of Pt 4f, the peak corresponding to CPB-4 at 70.1 eV was assigned to Pt 4f_7/2_, while another peak at 73.4 eV was ascribed to Pt 4f_5/2_.

It is widely known that the surface area strongly affects the adsorption and catalytic properties of photocatalysts. The N_2_ adsorption–desorption isotherms of Bi_2_MoO_6_, CdS, and CPB-4 are shown in Fig. 4. The BET specific surface areas of pure Bi_2_MoO_6_ and CdS were 16.13 and 18.25 m^2^ g^−1^, respectively. Compared with pure CdS, the loading of the Pt NPs onto the surface of CdS NRs slightly increased the surface area of 0.5 wt% Pt/CdS (21.06 m^2^ g^−1^). The high specific surface area of the ternary CPB-4 composites provided numerous active centers, improving the adsorption capacity and photocatalytic fuel denitrification activity. The ternary CPB-4 composites were fabricated by the assembly of the Bi_2_MoO_6_ NSs and Pt/CdS NRs, which displayed a larger surface area (48.46 m^2^ g^−1^) than other PCs. Pore size distribution of different samples further confirms that CPB-4 composites had mesoporous structure (Fig. S3b†). The presence of mesopores in CPB-4 favored multi-light scattering, which enhanced the capture of excitation light and thus enhanced photocatalytic activity.^22,50^

**Fig. 5:**
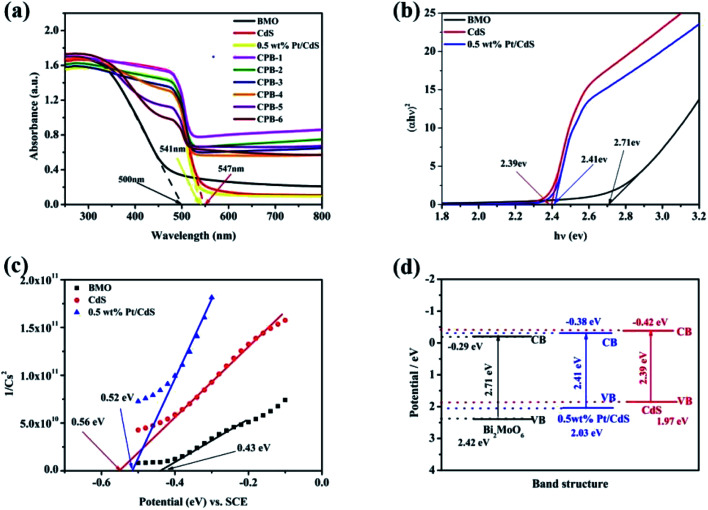
(a) UV-Vis diffuse reflectance spectra of CdS, Bi_2_MoO_6_, Pt/CdS, and CPB composites with different compositions. (b) Plots of (*αhv*)^2^*versus* (*hv*) used to estimate the optical band gaps of CdS, Pt/CdS, and Bi_2_MoO_6_. (c) Mott–Schottky plots of CdS, Pt/CdS, and Bi_2_MoO_6_. (d) Band structures of CdS, Pt/CdS, and Bi_2_MoO_6_.

The optical properties of the samples were evaluated by DRS. As shown in Fig. 5a, pure Bi_2_MoO_6_ and CdS displayed strong absorption peaks at 490 and 550 nm, respectively. Compared with pure CdS, the absorption of Pt/CdS did not change significantly in the ultraviolet and visible regions. This indicated that the deposition of Pt NPs resulted in the surface modification of the CdS NRs, rather than the replacement of the host Cd in the CdS lattice by Pt. More importantly, compared with pure Bi_2_MoO_6_, the visible-light absorption capacity of ternary CPB composites was significantly enhanced, suggesting that the constructed S-scheme HJ structure displayed a broadened light absorption range and improved photocatalytic activity. The UV-Vis absorption spectra of the low content Bi_2_MoO_6_ loaded on 0.5 wt% Pt/CdS further verified the above results (Fig. S4†). Moreover, the absorption wavelength of all ternary CPB composites exhibited a significant red shift. The band gap energy (*E*_g_) of the semiconductors was calculated according to the Kubelka–Munk function shown below (eqn (1)):

1 *αhv* = *A*(*hv* − *E*_g_)^*n*/2^

where *α*, *h*, *v*, and *A* refer to the absorption coefficient, Planck constant, light frequency, and a constant, respectively.^51^ Concurrently, *n* was related to the type of the optical transition in the semiconductor (*n* = 1 for direct transition, *n* = 4 for indirect transition). CdS and Bi_2_MoO_6_ were both determined as transition semiconductors; therefore, in this case, *n* = 1. The (*αhv*)^2^ and *hv* of Bi_2_MoO6, CdS, and Pt/CdS are summarized in Fig. 5b. Accordingly, the band gaps of pure Bi_2_MoO_6_, CdS, and Pt/CdS were established to be approximately 2.71, 2.39, and 2.41 eV, respectively.

The Mott–Schottky plots were used to further evaluate the band structures of pure Bi_2_MoO_6_, CdS, and Pt/CdS. As shown in Fig. 5c, the flat band potential values of Bi_2_MoO_6_, CdS, and Pt/CdS with respect to the saturated calomel electrode (SCE) were −0.43, −0.56, and −0.52 eV, respectively. Using the difference between the SCE and the standard hydrogen electrode, the conduction band positions of the samples were calculated to be −0.29, −0.42, and −0.38 eV (*vs.* NHE), respectively. The corresponding valence band positions of Bi_2_MoO_6_, CdS, and Pt/CdS were established to be 2.42, 1.97, and 2.03 eV, respectively, using eqn (2) (Fig. 5d).

2 *E*_VB_ = *E*_CB_ + *E*_g_

The positions of the conduction and valence bands of the composites clearly demonstrated that ternary CPB-4 could form closely coupled S-scheme HJs due to the well-matched band structures. An S-scheme HJ could provide a driving force for the charge transfer and significantly improved the photocatalytic activity of the composites.

**Fig. 6:**
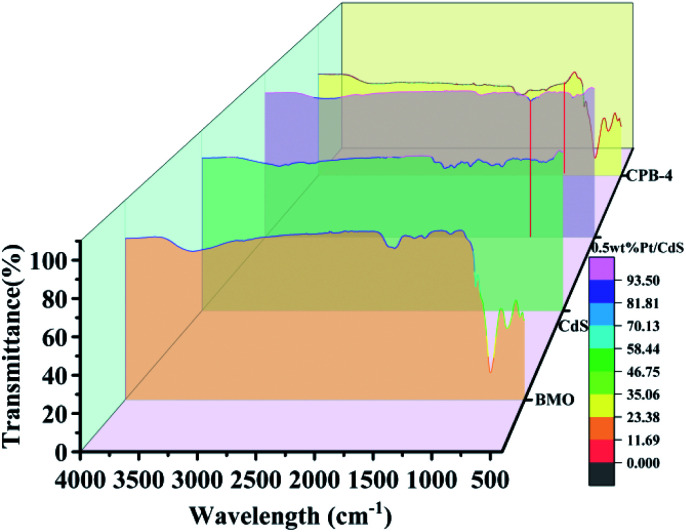
FT-IR spectra of Bi_2_MoO_6_, CdS, Pt/CdS, and CPB-4 composite.

The FT-IR spectra were subsequently investigated to confirm the effects of Bi_2_MoO_6_ and Pt on the CdS NRs interface as well as to analyze the characteristic chemical bonding in the prepared samples. As shown in Fig. 6, in the case of Bi_2_MoO_6_, the peaks at 707 and 831 cm^−1^ corresponded to the asymmetric and symmetric stretching vibration of the Mo–O bond, respectively. In addition, the signals at 450 and 538 cm^−1^ were attributed to biaxial stretching and bending vibration of the Bi–O bond,^52^ respectively. Compared to Bi_2_MoO_6_, the peaks of Mo–O and Bi–O in the spectrum of CPB-4 were sharper; however, the peak positions were unchanged. For both pure CdS and Pt/CdS NRs, the peaks of the CdS–S bonds were detected at 1110 and 1320 cm^−1^, correspondingly. Since the surfaces of both Pt/CdS and CPB-4 readily absorbed H_2_O, the characteristic peaks at approximately 1680 cm^−1^ were attributed to the O–H vibration. Notably, the FT-IR spectra of both Pt/CdS and CPB-4 exhibited absorption peaks at 1080 cm^−1^ due to the presence of Pt NPs, which enhanced the photocatalytic performance.

**Fig. 7:**
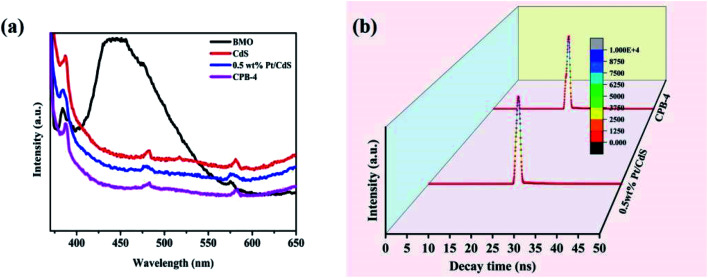
(a) PL spectra and (b) time-resolved photoluminescence decay spectra of Pt/CdS and CPB-4 composites.

Fig. 7a illustrates the PL spectra of Bi_2_MoO_6_, CdS, Pt/CdS, and the ternary CPB-4 composites. PL spectroscopy is frequently used to study the separation and transfer efficiency of photogenerated charge carriers. The lower the PL peak intensity of the photocatalyst, the lower the probability of the recombination of photoexcited electron–hole pairs. Bi_2_MoO_6_ and CdS showed strong PL peaks due to the relatively high recombination of the photogenerated electron–hole pairs. The PL intensity of Pt/CdS was weaker than that of CdS, indicating that the lifetime of the photogenerated carriers on Pt/CdS was longer. This was consistent with the fuel denitrification performance of Pt/CdS. Notably, the ternary CPB-4 composites displayed the lowest PL intensity among the studied catalysts, which implied that the S-scheme HJ exhibited efficient carrier separation ability. Moreover, the average lifetime of carriers in the photocatalysts was measured by transient fluorescence spectroscopy and calculated using the following formula (eqn (3)):

3 <*τ*> = (*A*_1_*τ*_1_^2^ + *A*_2_*τ*_2_^2^)/(*A*_1_*τ*_1_ + *A*_2_*τ*_2_)

where *τ*_1_ and *τ*_2_ indicate the decay lifetimes and *A*_1_ and *A*_1_ refer to the transient absorptions of excitons.^53^ The average lifetimes of Bi_2_MoO_6_, CdS, Pt/CdS, and CPB-4 were measured to be 0.35, 0.43, 0.39, and 0.40 ns, respectively (Fig. 7b). A significantly lower carrier lifetime was expected in the case of Pt/CdS than CdS. This is because when Pt acted as a cocatalyst on the surface of the CdS NRs, the closer the distance between the photogenerated holes and electrons transferred to the Pt NPs, the faster the recombination rate and the shorter the lifetime. The lifetime of the photogenerated carriers was enhanced due to the construction of S-scheme HJs from CPB-4.

### Photocatalytic activity

**Fig. 8:**
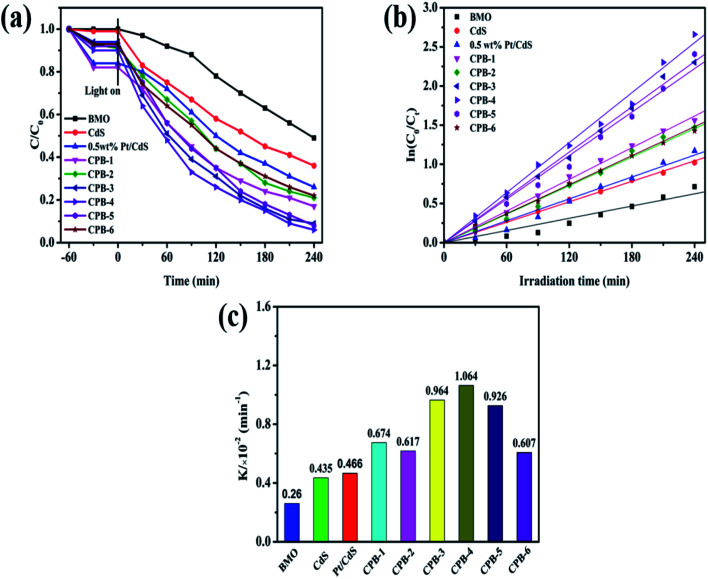
(a) Photocatalytic denitrogenation of pyridine under visible-light irradiation, (b) corresponding pseudo-first-order kinetics fitting curves of CPB composites with different proportions, (c) apparent rate constants over different catalysts.

The photocatalytic activities of all catalysts were evaluated by conducting photocatalytic denitrification of pyridine under visible-light irradiation (*λ* > 420 nm). As shown in Fig. S5,† the photodegradation efficiency of the CdS NRs was only 68% due to the significant carrier recombination in the bulk phase. Notably, 0.5 wt% Pt/CdS showed higher photocatalytic fuel denitrification activity than photocatalysts exhibiting other contents of Pt loaded on CdS NRs. This demonstrated that loading of an appropriate amount of the Pt NPs on the CdS NRs played a significant role in improving the photocatalytic performance. Based on Fig. 8a and Table 1, Bi_2_MoO_6_ displayed the lowest photocatalytic pyridine degradation performance among all of the studied photocatalysts. The CPB-4 composite exhibited excellent degradation performance with a pyridine degradation rate of 94% after visible-light irradiation for 240 min. The photodegradation activity of the CPB-4 composite was the highest among the ternary CPB materials, Pt/CdS, and two phase Bi_2_MoO_6_/CdS (Fig. S6†). To compare the photocatalytic degradation efficiency of all samples more accurately, the reaction kinetics of pyridine degradation were also examined (Fig. 8b). All kinetic data fit a straight line and followed a pseudo-first-order model: In(*C*_0_/*C*_T_) = *k*_app_*t*, where *t* is the irradiation time (min), while *C*_0_ and *C*_*t*_ are indicate the concentrations of pyridine. The kinetic rate constants of Bi_2_MoO_6_, CdS, Pt/CdS, CPB-1, CPB-2, CPB-3, CPB-4, CPB-5, and CPB-6 were determined to be 0.260, 0.435, 0.466, 0.674, 0.617, 0.964, 1.064, 0.926 and 0.607 (10^−2^ min^−1^), respectively. Fig. 8c clearly shows that the CPB-4 composite was the most active photocatalyst for the photocatalytic pyridine denitrification. Specifically, its activity was approximately 2.4 and 4.1 times higher than those of CdS and Bi_2_MoO_6_, respectively. The photocatalytic pyridine denitrification performance of all of the studied photocatalysts is summarized in Table 1. Moreover, Table 2 compares the catalytic activity of CPB-4 with other catalysts previously reported in the literature. Overall, compared with other photocatalysts, the ternary CPB-4 composites displayed the highest photocatalytic activity for pyridine denitrifcation under visible-light irradiation.

**Table 1:** Pyridine degradation rates and *K*_app_ values for all PCs

Sample	Denitrogenation efficiency (%)	*K*_app_ × 10^−2^ (min^−1^)
Bi_2_MoO_6_	51	0.260
CdS	68	0.435
0.5 wt% Pt/CdS	74	0.466
CPB-1	83	0.674
CPB-2	79	0.617
CPB-3	91	0.964
CPB-4	94	1.064
CPB-5	92	0.926
CPB-6	77	0.607

**Table 2:** Comparison the catalytic activity of CPB-4 with other PCs for denitrogenation of pyridine

Catalyst	*C*_pyridine_ (μg g^−1^)	*C*_cat_ (mg mL^−1^)	Light source	Time (h)	Efficiency (%)	Ref.
CPB-4	150	1	300 W (*λ* > 420 nm)	4	94	This work
TiO_2_/Fe_2_O_3_	100	1	300 W (*λ* > 420 nm)	4	92	59
Bi_20_TiO_32_	100	1	400 W (*λ* > 400 nm)	2.5	76	60
CeO_2_/TiO_2_	100	1	400 W (*λ* > 400 nm)	2.5	81	61
Pd/Bi_2_O_3_	100	1.5	300 W (*λ* > 420 nm)	1	84.9	10
Na(0.3)-C_3_N_4_	400	5	500 W (*λ* > 400 nm)	6	90	62
g-C_3_N_4_	130	5	500 W (*λ* > 400 nm)	6	91	62

**Fig. 9:**
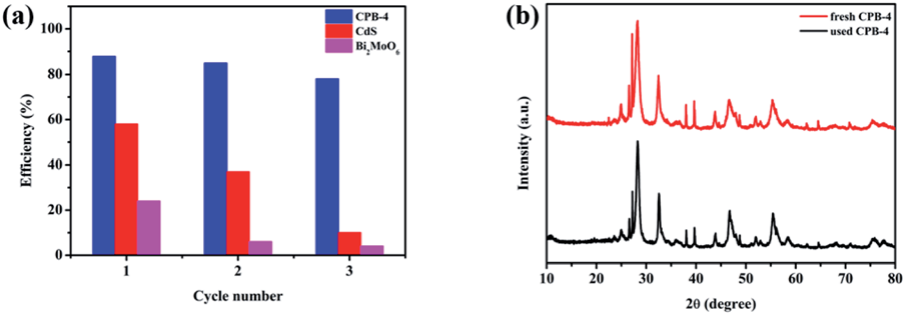
(a) Cycling experiments of photocatalytic denitrogenation of pyridine over CPB-4, (b) XRD patterns of fresh CPB-4 and used CPB-4 recovered after three cycles of photocatalysis.

The photocatalytic stability of Bi_2_MoO_6_, CdS, and CPB-4 was assessed by conducting cyclic photocatalytic pyridine denitrification reactions in the presence of the photocatalysts. As shown in Fig. 9a, CPB-4 exhibited stable photocatalytic activity even after three cycles of pyridine denitrification. Notably, the degradation rate remained above 80%. In contrast, the pyridine denitrification activities of Bi_2_MoO_6_ and CdS were unstable, and the degradation rate became low after three cycles (∼4% and ∼10%, respectively). It may be that CdS underwent its own photocorrosion, which in turn caused the photocatalytic stability to become poor. The excellent stability and efficient degradation ability of the ternary CPB-4 composites were attributed to the formation of an S-scheme between Bi_2_MoO_6_ and Pt/CdS, which improved the separation of the charge carriers and resulted in the generation of more active species that participated in the degradation reaction. Furthermore, the XRD patterns of the fresh and used CPB-4 composites are illustrated in Fig. 9b. The XRD results revealed that the characteristic peaks of the CPB-4 composites did not exhibit any significant changes after three cycles. This demonstrated that the CPB-4 composites were efficient, easy to prepare, and structurally stable and could be effectively employed as photocatalysts for the denitrification of fuel under visible light. Moreover, further SEM characterizations were conducted on the fresh CPB-4 and recovered CPB-4 samples, which showed no difference in structure and both possessed the structure of CdS NRs and Bi_2_MoO_6_ NSs (Fig. S8†).

### Photocatalytic mechanism

**Fig. 10:**
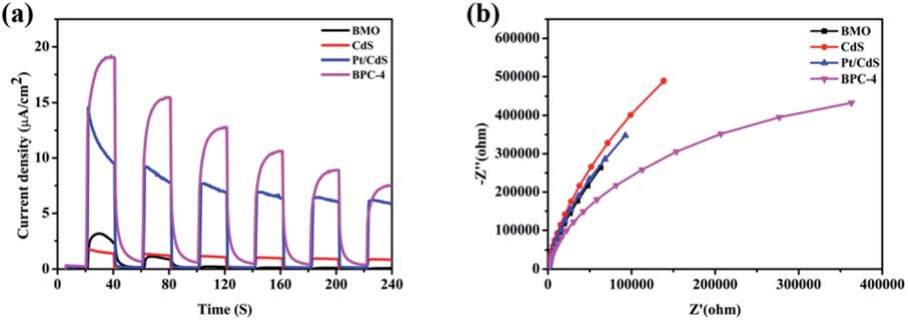
(a) Transient photocurrent responses and (b) EIS analysis of CdS, Bi_2_MoO_6_, Pt/CdS, and CPB-4 composites.

The rate of carrier separation and recombination is an important factor affecting the performance of photocatalysts. Fig. 10a shows the transient photocurrent responses of Bi_2_MoO_6_, CdS, Pt/CdS, and CPB-4. The photocurrent response of pure Bi_2_MoO_6_ was very weak, which was ascribed to the rapid recombination of photogenerated carriers. Compared with pure CdS, Pt/CdS possessed four times transient photocurrent density. Among all of the examined photocatalysts, the CPB-4 composites exhibited the highest photocurrent response, indicating that the combination of Bi_2_MoO_6_ and Pt/CdS improved the interfacial charge transfer efficiency. In addition, the carrier migration and transfer processes of the photocatalysts were investigated by electrochemical impedance spectroscopy (EIS). The CPB-4 composites showed the smallest arc radius among all of the analyzed photocatalysts (Fig. 10b). This indicated that the CPB-4 composites displayed the lowest resistance among all of the samples and that the recombination of electrons and holes at the interface was suppressed. Hence, the S-scheme HJ formed by Bi_2_MoO_6_ and Pt/CdS could effectively promote the separation and transfer of electrons and holes, improving the photocatalytic fuel denitrification activity. Furthermore, HPLC-MS and ESR spectroscopy were employed to investigate the mechanism of fuel denitrification in detail. The simulated fuel was monitored under visible-light irradiation using HPLC-MS for 0 and 4 h (Fig. S7†). After 4 h of visible-light irradiation, the intensity of the peak corresponding to pyridine (*m*/*z* = 79.8) considerably decreased, indicating successful degradation of the compound.

**Fig. 11:**
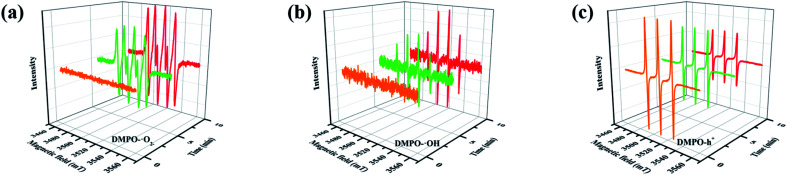
(a–c) Electron spin response spectra of various radical adducts.

**Fig. 12:**
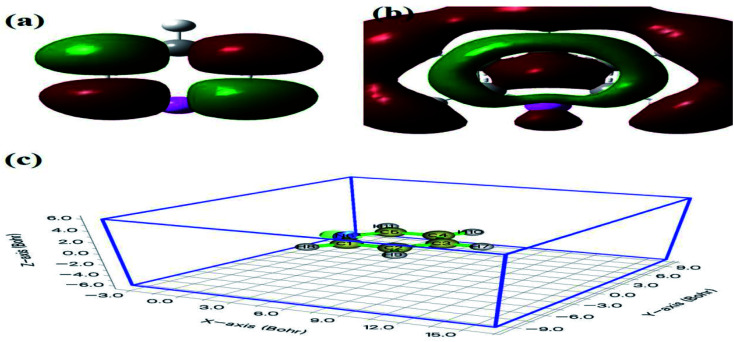
Pyridine molecular frontier orbital of (a) HOMO, (b) LUMO, and (c) the Fukui function value.

**Fig. 13:**
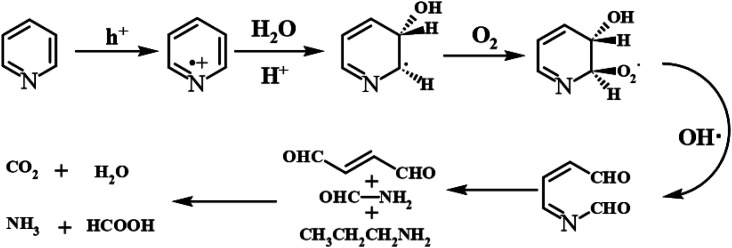
The denitrification pathway of pyridine.

Furthermore, compared with the spectrum obtained following visible-light irradiation for 0 h, after 4 h, three new peaks were detected at *m*/*z* = 46.0, 59.0, and 85.9, suggesting that pyridine was transformed into NH_2_CHO, CH_3_(CH_2_)NH_2_, and C_4_H_4_O_2_, which were the protonated intermediates. Moreover, as shown in Fig. 11, ESR was used to detect the dynamic changes of free radicals in the pyridine denitrification system *in situ*. DMPO is commonly employed for trapping hydroxyl radicals (˙OH) and superoxide radicals (˙O_2_^−^) to generate the corresponding DMPO–˙OH and DMPO–˙O_2_^−^ compounds.^26^ As shown in Fig. 11a and b, no signals attributed to the free radicals were detected under dark conditions. However, after 5 min of visible-light irradiation, characteristic tetragonal peaks of DMPO–˙OH and DMPO–˙O_2_^−^ were easily detected. It is noteworthy that the signals obviously increased with increasing light irradiation time, confirming the generation of ˙OH and ˙O_2_^−^ during the degradation of pyridine by light irradiation. The valence band potential of CPB-4 was more positive than that of *E*_(˙OH/H_2_O)_ (2.27 eV *vs.* NHE).^54^ Thus, the generation of ˙OH radicals by H_2_O oxidation was thermodynamically achievable. Similarly, the conduction band potential of CPB-4 was more negative than that of *E*_(˙O2^−^/O_2_)_ (−0.33 eV *vs.* NHE), demonstrating that the oxygen in the air combined to generate ˙O_2_^−^ in the system. In addition, we also examined the active hole (h^+^) species. TEMPO is generally regarded as a hole probe because its radicals can be oxidized by holes. As illustrated in Fig. 11c, the characteristic signal attributed to TEMPO was strong under dark conditions and significantly decreased with increasing irradiation time. This confirmed the photogeneration of holes during the degradation of pyridine. Hence, based on our analysis, it was determined that ˙OH, ˙O_2_^−^, and h^+^ all participated in the photocatalytic pyridine denitrification reaction. In addition, the presence of these three active species considerably enhanced the efficiency of pyridine denitrification. We also conducted theoretical calculations of the reaction sites for the fuel denitrification system using molecular frontier orbitals and Fukui functions. The distribution of frontier orbitals, *i.e.*, the highest occupied molecular orbital (HOMO) and the lowest unoccupied molecular orbital (LUMO), is frequently used to explain the reaction directions of organics molecules.^55,56^Fig. 12a and b shows the distribution of HOMO and LUMO on the N atom of pyridine. Moreover, the Fukui function is a commonly employed real space function for predicting reaction sites. In the present study, the isosurface plots of the Fukui function were calculated for the pyridine system. As illustrated in Fig. 12c, the isosurface only appeared on the N atoms. This revealed that the value of the Fukui function near the carbon atom was less than that on the N atom, implying that the pyridine degradation reaction would preferentially occur on the latter, which was consistent with the analysis of the molecular frontier orbitals. Based on the LC-MS, ESR, and theoretical analyses, we proposed a plausible pyridine denitrification pathway (Fig. 13).

**Fig. 14:**
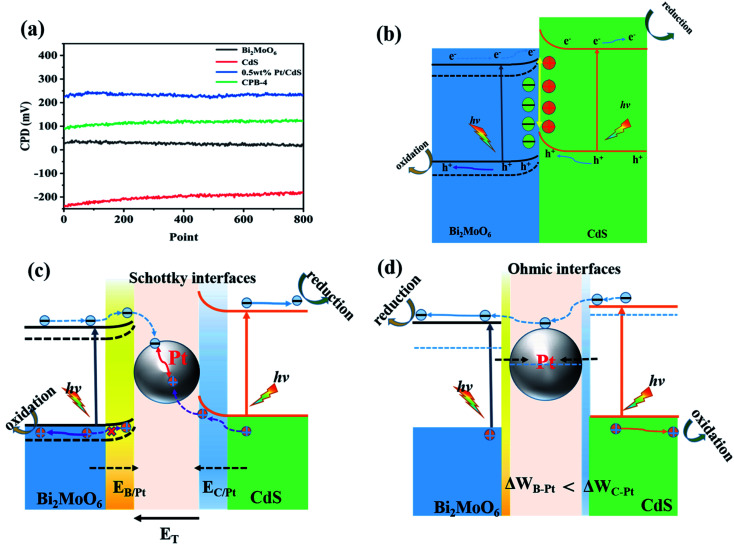
(a) CPDs values relative to Au at the photocatalyst surface, (b) CdS/Bi_2_MoO_6_ S-scheme heterojunction systems, and electronic structure models and charge transfer under S-scheme heterojunction systems of (c) Schottky contacts and (d) ohmic contact.

**Scheme 2:**
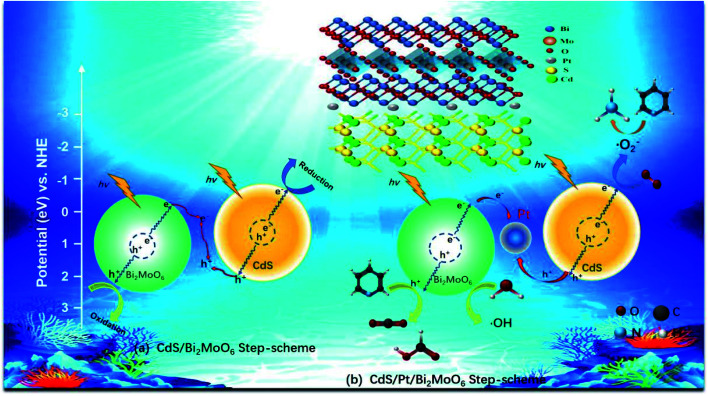
S-scheme mechanism of photocatalytic denitrogenation of pyridine of (a) CdS/Bi_2_MoO_6_ and (b) CdS/Pt/Bi_2_MoO_6_ composite.

We elucidated the mechanism of the CdS/Bi_2_MoO_6_ S-scheme HJ. Since the work function of Bi_2_MoO_6_ (*W*_B_ = 5.126 eV) was larger than that of CdS (*W*_C_ = 4.899 eV) (Fig. 14a), when the two phases came into contact, the electrons were spontaneously transferred from CdS to Bi_2_MoO_6_ at the interface. This led to the formation of a positive charge on one side of CdS and a negative charge on one side of Bi_2_MoO_6_, resulting in an equilibrium and the generation of a built-in electric field^29^ (Fig. 14b). Under light irradiation, the electrons of Bi_2_MoO_6_ and CdS were excited to the conduction band. Under the effect of the built-in electric field, the photogenerated electrons in the conduction band of Bi_2_MoO_6_ and the photogenerated holes of CdS were recombined on the interface to form an S-scheme HJ. A similar mechanism was proposed for the ternary CPB photocatalytic system (Scheme 2). Following the introduction of Pt NPs into CdS/Bi_2_MoO_6_ (Fig. 14c), ternary CPB-4 consisted of two tandem Schottky junctions (CdS/Pt–Pt/Bi_2_MoO_6_). Notably, both CdS/Pt and Pt/Bi_2_MoO_6_ were Schottky contacts. Due to the generation of the built-in electric field, under dark conditions, the bands corresponding to CdS and Bi_2_MoO_6_ were bent upward at the interface of CdS/Pt and Pt/Bi_2_MoO_6_.^57^ Under visible-light irradiation, the electrons were excited from the valence band of Bi_2_MoO_6_ to the conduction band of CdS, while the holes remained in the valence band. According to the Schottky effect model, the photogenerated holes in Bi_2_MoO_6_ and CdS were transferred to the Pt NPs under the influence of the built-in electric field. It is noteworthy that the built-in electric field at the CdS/Pt interface was stronger than that at the Pt/Bi_2_MoO_6_ interface (*W*_C_ < *W*_B_). Furthermore, the photogenerated holes on CdS preferentially migrated to the Pt NPs, compounding with the photogenerated electrons from the conduction band of Bi_2_MoO_6_, which more easily crossed the Schottky energy barrier on the surface of the Pt NPs (Fig. 14c). Considering the model of the ohmic contact shown in Fig. 14d, since the work function difference between Pt and CdS was larger than that between Pt and Bi_2_MoO_6_ (Δ*W*_C-Pt_ > Δ*W*_B-Pt_), the photogenerated electrons tended to migrate from CdS to the Pt NPs rather than from Bi_2_MoO_6_ to the Pt NPs.^58^ Evidently, ˙OH and ˙O_2_^−^ would not be generated by this charge migration mode, which was inconsistent with the ESR characterization results. In the CPB-4 S-scheme HJ structure, the Pt NPs acted as a “bridge,” enabling fast combination of the electrons from the conduction band of Bi_2_MoO_6_ with the holes from the valence band of CdS by transport channels. Hence, the photogenerated electrons and holes in the conduction and valence bands of CdS (2.42 eV *vs.* NHE) and Bi_2_MoO_6_ (−0.42 eV *vs.* NHE), respectively, were retained. This indicated that the CPB-4 S-scheme HJ developed in this work was able to efficiently separate charge carriers. Moreover, its excellent fuel denitrification performance could be attributed to the strong redox capability retained by its S-scheme HJ.

## Conclusions

In this work, a novel S-scheme CdS/Pt/Bi_2_MoO_6_ HJ photocatalyst was prepared by ultrasonic deposition and hydrothermal methods. When the molar ratio of Bi_2_MoO_6_ to Pt/CdS was 2 : 5, the developed ternary CPB-4 S-scheme HJ photocatalyst exhibited excellent photocatalytic activity (up to 94%) for fuel denitrification under visible-light irradiation for 4 h. The pyridine degradation rate of CPB-4 was 0.01064 min^−1^, which was 2.4 and 4.1 times higher than that of CdS (0.00436 min^−1^) and Bi_2_MoO_6_ (0.00260 min^−1^), respectively. Moreover, after three pyridine denitrification cycles, CPB-4 retained more than 80% activity, indicating good stability and recyclability of the composite. When the Pt NPs were anchored on the CdS NRs, exerting a role of bridges, the recombination of unwanted photogenerated electrons and holes was enhanced. The introduction of Pt NPs and Bi_2_MoO_6_ NSs into the S-scheme HJ system promoted the absorption of visible light and greatly increased the fuel denitrification performance. The present study describes a novel concept of introducing Pt NPs into S-scheme HJ for efficient photocatalytic fuel denitrification and stable photocatalyst recycling. The outcomes of this work will undoubtedly lead to the design of new S-scheme HJ structures. Importantly, the present study provides a general approach for highly effective degradation of environmental pollutants, specifically NCCs.

## Conflicts of interest

There are no conflicts of interest to declare.

## Supplementary Material

RA-011-D1RA04417F-s001
