# Supplementary material for: Construction of a novel step-scheme CdS/Pt/Bi2MoO6 photocatalyst for efficient photocatalytic fuel denitrification
Source: RSC Adv. 2021 Jul 1;11(38):23288–300. doi: 10.1039/d1ra04417f (PMC9036592; doi:10.1039/d1ra04417f)
Supplement: RA-011-D1RA04417F-s001 [file RA-011-D1RA04417F-s001.pdf]

Electronic Supplementary Material (ESI) for RSC Advances.
This journal is © The Royal Society of Chemistry 2021

Supplementary information

Construction of Novel Step-scheme CdS/Pt/Bi_2MoO_6 Photocatalyst for Efficient Photocatalytic Fuel Denitrification

Weineng Hu[1,2,3], Guiyang Yan[1,2,*], Ruowen Liang[1,2], Mengmeng Jiang[3], Renkun Huang[1,2], Yuzhou Xia[1,2], Lu Chen[1,2], and Yi Lu[1,2]

[1] Department of chemistry, Fujian province university key laboratory of green energy and environment catalysis, Ningde Normal University, Ningde 352100, P. R. China

[2] Department of chemistry, Fujian Provincial Key Laboratory of Featured Materials in Biochemical Industry, Ningde Normal University, Ningde 352100, Fujian, China

[3] State Key Laboratory of Photocatalysis on Energy and Environment, Fuzhou University, Fuzhou 350002, P. R. China

* Corresponding author: Prof. Guiyang Yan, E-mail: ygyfjnu@163.com

Supporting Figures and Table

1、Details of characterization

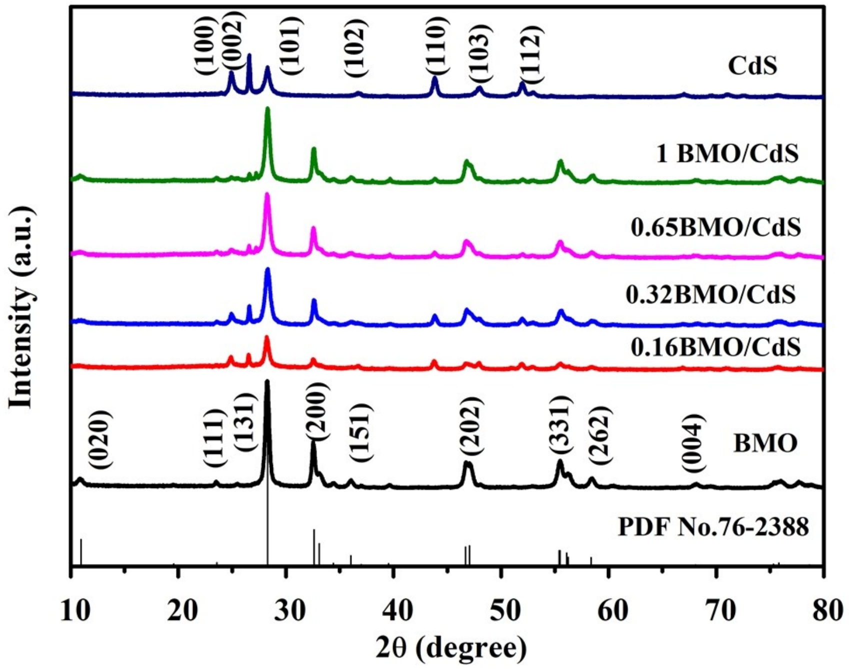

Fig. S1. XRD patterns of a series of Bi_2MoO_6/CdS.

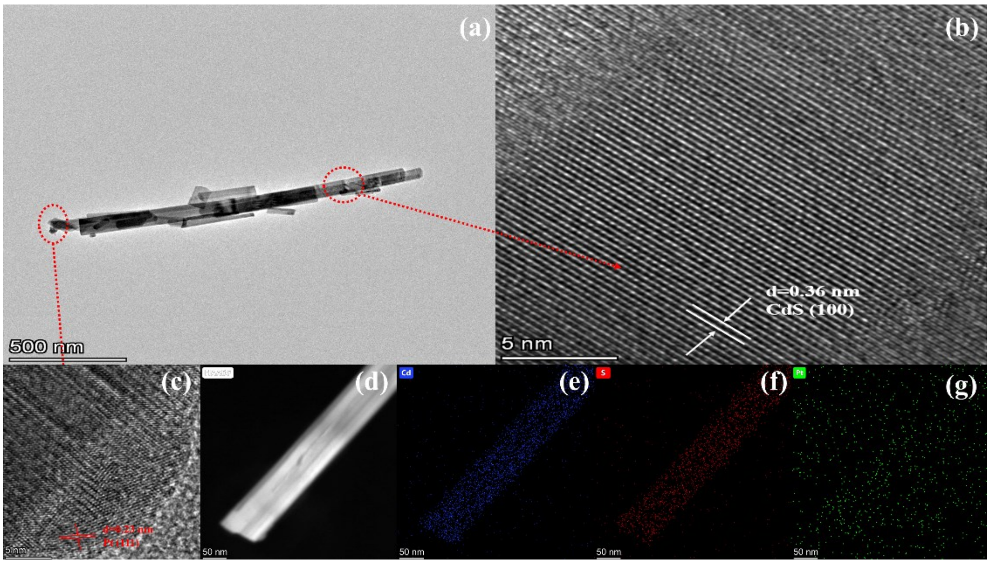

Fig. S2. Typical TEM images of (a) Pt/CdS, (b,c) HRTEM images of Pt/CdS and (d-g) element mapping images of Pt/CdS.

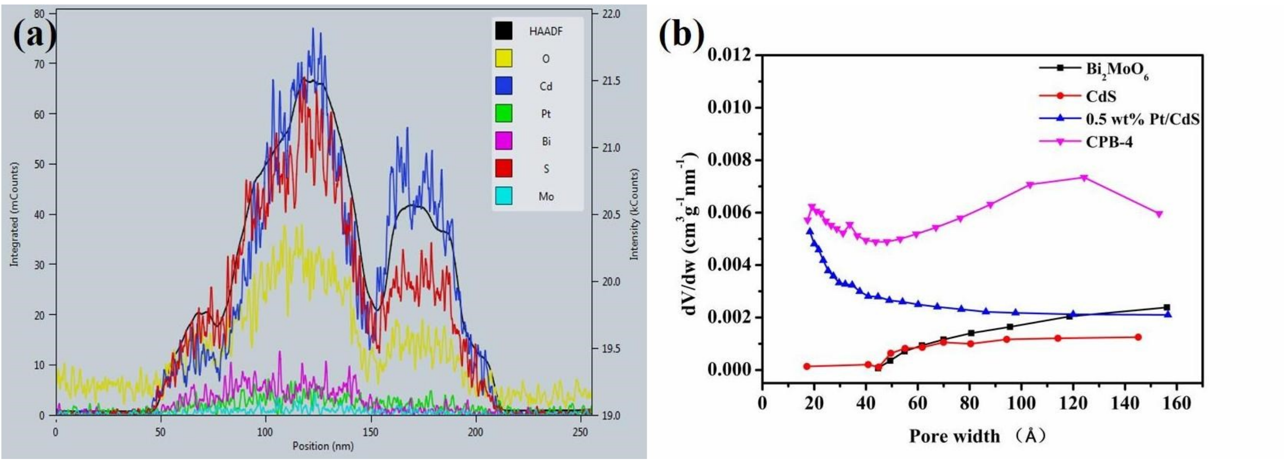

Fig. S3. (a) EDS of CPB-4 photocatalysts and (b) pore size distribution curves of photocatalysts

As prepared CdS/Pt/Bi_2MoO_6 composites (x wt% Bi_2MoO_6 vs. CdS/Pt) with mass ratios of 10%, 5%, 2.5% and 1% are labeled as CPB-1S, CPB-2S, CPB-3S and CPB-4S, respectively.

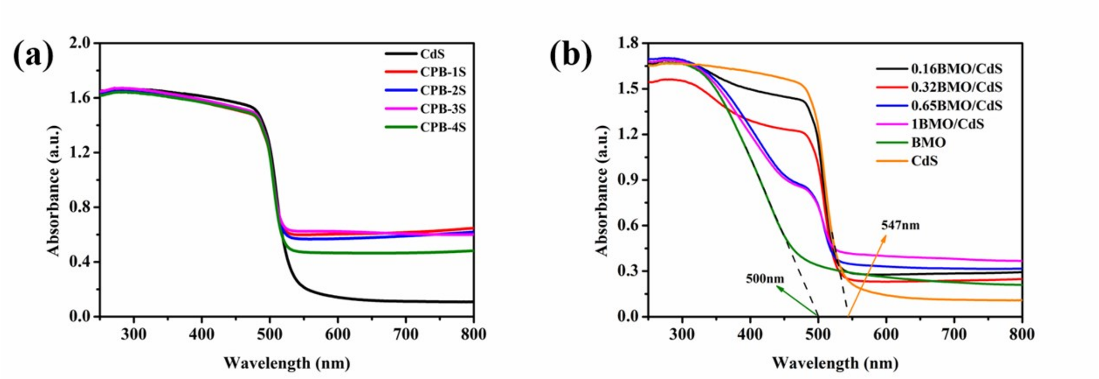

Fig. S4. (a) UV-vis diffuse reflectance spectra of CdS, and CPBS (trace amounts of Bi_2MoO_6) with different compositions and (b) UV-vis diffuse reflectance spectra of Bi_2MoO_6/CdS.

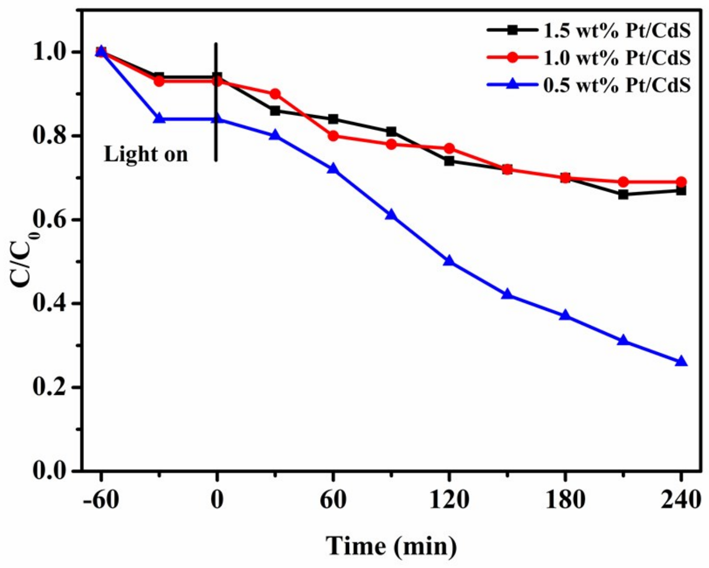

Fig. S5. Photocatalytic denitrogenation of pyridine under visible-light irradiation of other ratios of Pt loaded CdS NRs

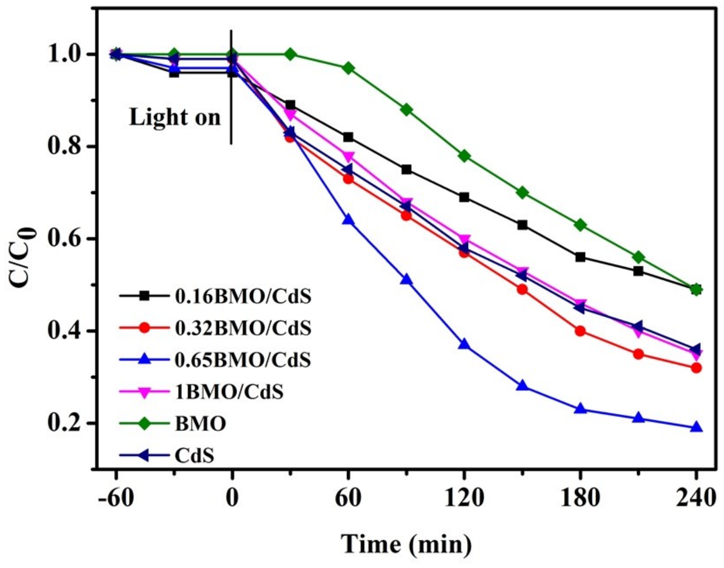

Fig. S6. Photocatalytic denitrogenation of pyridine under visible-light irradiation.

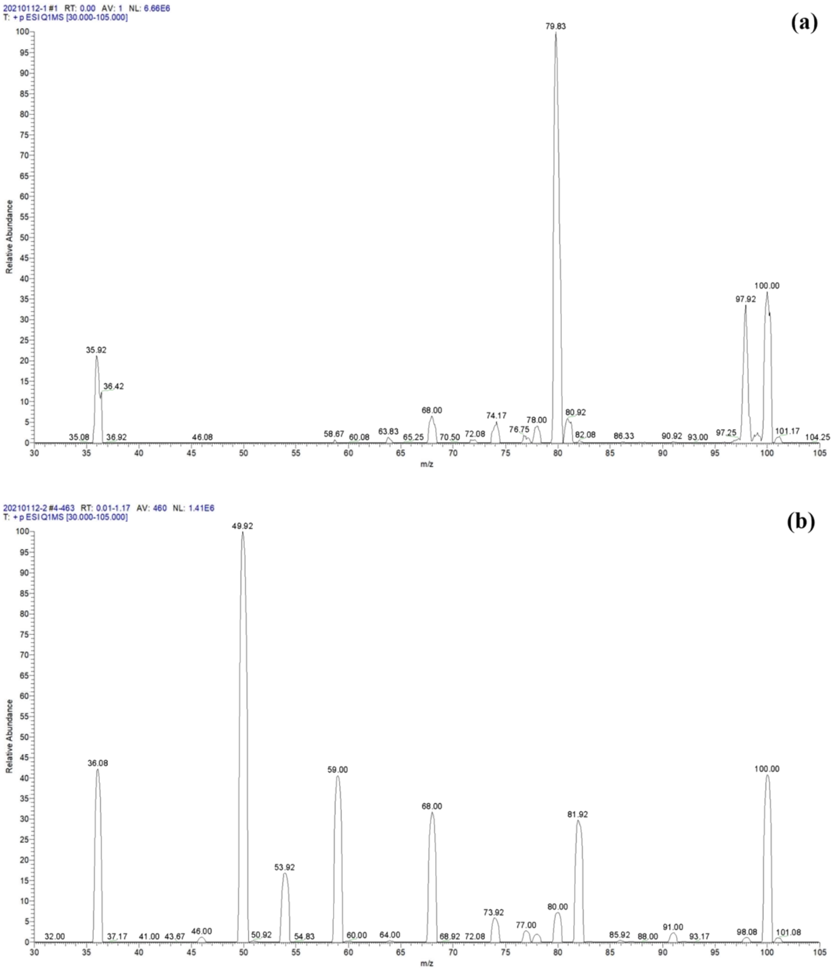

Fig. S7. High-performance liquid chromatography profiles of pyridine after different irradiation times: (a) 0h, and (b) 4h

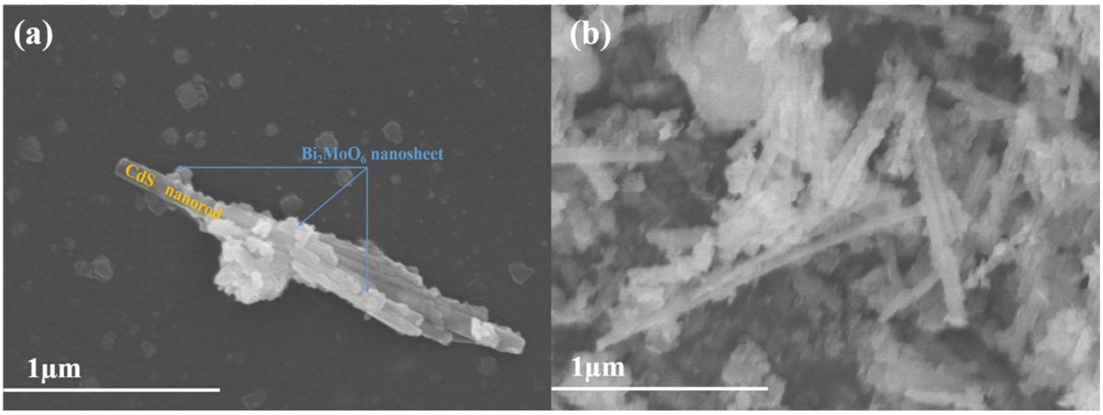

Fig. S8. SEM images of (a) Initial CPB-4 and (b) CPB-4 after three cycles

2、The details of the fuel denitration cycle

After the first experiment, the photocatalyst was collected into a centrifuge tube, separated by centrifugation (8000 rpm,6 min), washed several times with deionised water and ethanol, and then were dried in a vacuum drying oven at 80°C for 6 h. Afterward, the produced yellow-green solids were collected. Then it was the same as the first pyridine removal experiment and the concentration was detected after 4 hours of visible light irradiation (>420 nm).

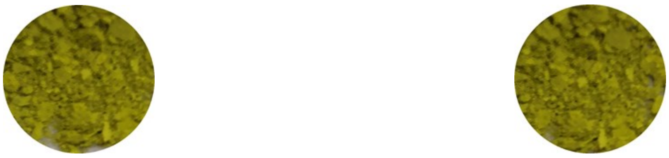

Fig. S9. (a) Initial CPB-4, (b) photographs of samples of CPB-4 after three cycles.
